# Predictive Value of Basal Serum Progesterone for Successful IVF in Endometriosis Patients: The Need for a Personalized Approach

**DOI:** 10.3390/jpm12101639

**Published:** 2022-10-03

**Authors:** Jovan Bila, Jelena Dotlic, Svetlana Spremovic Radjenovic, Snezana Vidakovic, Lidija Tulic, Jelena Micic, Jelena Stojnic, Ivana Babovic, Aleksandar Dmitrovic, Vito Chiantera, Antonio Simone Laganà, Milan Terzic

**Affiliations:** 1 Clinic of Obstetrics and Gynecology, University Clinical Centre of Serbia, Dr Koste Todorovica 26, 11000 Belgrade, Serbia; 2 Faculty of Medicine, University of Belgrade, Dr Subotica Starijeg 8, 11000 Belgrade, Serbia; 3 Unit of Gynecologic Oncology, ARNAS “Civico-Di Cristina-Benfratelli”, Department of Health Promotion, Mother and Child Care, Internal Medicine and Medical Specialties (PROMISE), University of Palermo, 90127 Palermo, Italy; 4 Department of Medicine, School of Medicine, Nazarbayev University, Zhanybek-Kerey Khans Street, 5/1, Nur-Sultan 010000, Kazakhstan or; 5 Clinical Academic Department of Women’s Health, National Research Center for Maternal and Child Health, Corporate Fund “University Medical Center”, Turan Ave. 32, Nur-Sultan 010000, Kazakhstan; 6 Department of Obstetrics, Gynecology and Reproductive Sciences, University of Pittsburgh School of Medicine, 300 Halket Street, Pittsburgh, PA 15213, USA

**Keywords:** progesterone, endometriosis, surgery, in vitro fertilization, personalized approach

## Abstract

The data regarding the role of progesterone (P4) in reproductive events of endometriosis patients are limited. This prospective study aimed to examine the predictive value of basal P4 serum levels for successful in vitro fertilization (IVF) in patients with primary infertility and endometriosis. The study included 73 patients divided according to endometriosis treatment (surgery vs. control—no treatment). The general data, basal hormonal status, and pregnancy rates were determined for every patient. Clinical pregnancy was achieved in 40.3% of patients, and more often in patients treated for endometriosis before IVF. The regression analysis showed that higher basal P4 serum levels were associated with achieving pregnancy through IVF. When regression was adjusted for the patient and IVF characteristics, higher basal P4 serum levels were associated with pregnancy achievement in both groups of women, along with the basal serum levels of FSH, LH, and AMH; EFI score; and stimulation protocol. The ROC analysis showed that the basal P4 serum level for successful IVF should be ≥0.7ng/mL. The basal P4 serum level cut-off for IVF success in endometriosis patients was determined for the first time. Constructed models for IVF success prediction emphasize the importance of determining the basal P4 serum levels for the personalized treatment of endometriosis-related infertility.

## 1. Introduction

Endometriosis is a chronic and progressive estrogen-dependent disease, characterized by the presence of endometrial-like tissue, glands, and stroma outside the uterine cavity. To date, its pathogenesis remains to be fully elucidated, and available data about the reproductive prognosis in this population, with or without IVF, are still scarce. Several studies show that it occurs in the female general population with a prevalence of around 10%, and it is most common in women of the reproductive age. Infertility affects 30% to 50% of women with endometriosis [1,2]. Infertility treatment in patients with endometriosis is often associated with a diverse pattern of success, underlining the need for a personalized approach, taking into consideration individual patient characteristics. The tendency to improve the outcome should start with an evaluation of the parameters that could help with perceiving and predicting treatment outcomes. However, to date, there are not many pieces of evidence that could suggest the predictive value of basal hormone levels in the evaluation of these patients. The most commonly used endocrinologic indicators of treatment success are follicle-stimulating hormone (FSH), anti-Müllerian hormone (AMH), and progesterone (P4) levels [3,4,5], although no specific hormonal parameter for women affected by endometriosis is currently available to predict fertility prognosis. 

Patients with endometriosis have altered progesterone signaling mechanisms and general progesterone receptor deficiency [6,7]. In particular, the altered expression of progesterone receptors plays a key role in both ectopic and eutopic endometrium, causing progesterone resistance, which may underlie, at least in part, impaired fertility outcomes in women affected by endometriosis [8,9,10]. Moreover, endometriosis tissue shows an overexpression of estradiol (E2) receptor alpha, which is positively correlated with more advanced stages of endometriosis, leading to increased E2 production and secretion, mainly caused by the increased expression of aromatase within endometriosis foci [5,6]. This altered E2/P4 ratio, besides its impact on the endometrium, can negatively affect the menstrual cycle, folliculogenesis, quality of oocytes, and, consequently, even the success rate of in vitro fertilization (IVF) [8,9,10]. Nevertheless, clinical research on P4 and IVF success in patients with endometriosis is limited. Moreover, it would be important to assess whether the basal P4 determination in patients with endometriosis would lead to individually tailored approaches in ovarian stimulation.

For these reasons, we conducted a study with the primary aim to evaluate the predictive value of the basal P4 serum levels for successful IVF procedures and, as a secondary aim, to identify a potential subpopulation of women with primary infertility and endometriosis that may need individualized IVF pretreatment based on the P4 serum levels.

## 2. Materials and Methods

The study was performed at the Clinic for Obstetrics and Gynecology University Clinical Center of Serbia over a period of five years. It prospectively included women with primary infertility caused by endometriosis that were scheduled for IVF. 

The design, analysis, interpretation of data, drafting, and revisions conformed to the Helsinki Declaration, the Committee on Publication Ethics (COPE) guidelines (http://publicationethics.org; accessed on 1 February 2022), and the REporting of studies Conducted using Observational Routinely-collected health Data (RECORD) Statement, available through the Enhancing the Quality and Transparency of Health Research (EQUATOR) network (www.equator-network.org; accessed on 1 February 2022). The data collected were anonymized, taking into account the observational nature of the study, without personal data that could lead to the formal identification of the patient. Each patient enrolled in this study was informed about the study procedures, and signed consent to allow for data collection and analysis for research purposes. The study was approved by the Ethical Committee of the Faculty of Medicine University of Belgrade, Serbia, (Institutional Review Board Approval-University of Belgrade, number 61206-2616/2-2013). The study was not advertised. No remuneration was offered to the patients in order to give consent to be enrolled in this study.

The study inclusion criteria were being ≤40 years old, primary infertility caused only by endometriosis, no other genital pathology or infertility factors, body mass index (BMI) ≤ 30 kg/m^2^, regular cycles 25–35 days, and adequate basal ovarian reserve (3–15 antral follicles per ovary). Women affected by both ovarian and/or peritoneal endometriosis were eligible for the study. On the contrary, the exclusion criteria were age > 40 years, BMI > 30 kg/m^2^, secondary infertility, menstrual cycle disorders, associated infertility factors, malignancy, or any other genital pathology (pelvic inflammatory disease, myoma, other ovarian cysts, etc.).

The included women were divided into two groups based on endometriosis treatment. In the study, Group I (GI) included patients surgically and medically treated for endometriosis, while Group II (GII) was the control group with patients directly addressed to the IVF cycles. All women from the GI were treated according to the same protocol, which included surgery as the first line therapy, followed by GnRH agonists every 28 days for 6 months after surgery. Women were included in the GI according to the current recommendations for surgical treatment, which comprise having moderate to severe endometriosis (ASRM III/IV) in the pelvis or having an endometrioma diameter ≥ 3 cm confirmed upon diagnostic laparoscopy. On the contrary, women with minimal to mild endometriosis (ASRM I/II) in the pelvis and with an endometrioma < 3 cm were submitted to IVF without prior surgical treatment (GII). 

Data from personal and past medical history, such as age, body mass index (BMI), and infertility duration, were taken from all participating patients. Moreover, we determined the basal hormonal status (FSH, LH, E2, P4, and AMH) of all of the patients.

The levels of follicle-stimulating hormone (FSH), luteinizing hormone (LH), estradiol (E2), and progesterone (P4) were determined for all patients on the second or third day of their menstrual cycle, before starting any ovulation stimulation, so as anti-Mullerian hormone (AMH). Blood samples were taken with Vacutainer tubes (BD Vacutainer Systems) and were centrifuged according to the manufacturer’s instructions in order to obtain the serum samples. The chemiluminescent immunoassay (ECLIA) method was used to examine the basal levels of FSH, LH, E2, and P4, and it was performed on an Access 2 immunoassay system, Beckman Coulter. The FSH levels were expressed in IU/l, with reference levels for the follicular phase of 3.5–12.5 IU/l and for the mid-cycle of 4.7–21.5 IU/l. The LH levels were expressed in IU/l, with reference levels for the follicular phase of 2.4–12.6 IU/l and for the mid-cycle of 14–95.6 IU/l. An FSH/LH ratio was determined for each patient. Estradiol levels were expressed in pg/mL, with reference levels for the follicular phase of 12.3–232.7 pg/mL and for the mid-cycle of 41.1–400 pg/mL. P4 levels were expressed in ng/mL, with reference levels for the follicular phase of 0.31–1.52 ng/mL and for the luteal phase of 5.16–18.56 ng/mL. The serum AMH levels were determined by ELISA (enzyme-linked immunosorbent assay); expressed in ng/mL; and defined in interval ranges of low (<1 ng/mL), normal (1–5 ng/mL), and high (>5 ng/mL).

The women primarily had a thorough gynecological and ultrasound examination with uterine and endometrial assessment, antral follicles counting, and detecting the presence, diameter, and laterality of endometriomas. In addition, laparoscopy was performed in all cases, either only for endometriosis diagnostics or for treatment as well. We used the guidelines of the American Society of Reproductive Medicine (ASRM) to diagnose and stage endometriosis. Upon laparoscopy, the endometriosis fertility index (EFI) and the ASRM endometriosis stage were calculated. History data, such as age, parity, duration of infertility, and anatomical–functional assessment of disease severity during surgery, are included in the Endometriosis Fertility Index or EFI score, which is used to assess fertility after the surgical treatment of endometriosis. If the EFI score is ≥5 after 12 months from surgery, patients should be referred to IVF [11,12].

Surgical treatment for women from the study GI implied ovarian cystectomy and/or excision/vaporization of the pelvic endometriosis foci, with adhesiolysis where indicated. The stripping technique was used with meticulously bipolar diathermy by an experienced laparoscopic surgeon. Tissue samples were taken from the lesions and were histologically analyzed, thus confirming endometriosis. 

The controlled ovarian hyperstimulation (COH) was performed according to the following three protocols: the long protocol with the GnRH agonist, the short protocol with the GnRH agonist, and the short protocol with the GnRH antagonist. The long protocol implied pituitary suppression with the GnRH agonists at a dose of 0.1 mg per day, 7 days before the onset of the cycle, and it was continued daily until the end of ovulation stimulation. The short protocol implied pituitary suppression with the GnRH agonist, triptorelin, at a dose of 0.1 mg per day, starting from the second or third day of the cycle, daily, until the end of the stimulation of ovulation. The short protocol with the GnRH antagonist implied the usage of the use of the GnRH antagonist at a dose of 0.25 mg per day, from the sixth day of stimulation, and it was continued daily until the end of the stimulation. Ovarian stimulation started on the second or the third day of the cycle and it was conducted by giving subcutaneous injections of FSH (follitropin α) and/or HMG (menotropin) on a daily basis, with starting dose of 300 I.U. The ovarian stimulation was monitored through determination of the serum E2 and LH levels, as well as by transvaginal ultrasound monitoring of the follicular growth and endometrium thickness, and through homogenous triple layer appearance every second day from the sixth day of the cycle. When the E2 levels were above 400 pg/mL per follicle and there were at least two follicles greater than 18 mm, human chorionic-gonadotropin (HCG) was administered at a dose from 5000 to 10,000 IU. Follicular and oocyte aspiration were performed through transvaginal ultrasound control 34 to 36 h after the administration of HCG. The selection of the protocols depended on the patient’s age; EFI, FSH, E2, and AMH serum levels; and antral follicle count (AFC). Long protocol was applied in the case of a higher endometriosis stage, EFI > 7, FSH > 10 IU/L, low AMH < 1 ng/mL, and AFC < 5; otherwise, the short protocol was used. The short antagonist protocol was applied in patients with basal LH > 6 IU/L. In all other cases, ovulation was stimulated with the short agonist protocol. 

Ovarian response to stimulation was evaluated according to the number of retrieved oocytes (poor ≤ 4; adequate 5–15; excessive > 15 oocytes). The total number of embryos and their quality was assessed by three expert embryologists together. Based on the European Society of Human Reproduction and Embryology (ESHRE) recommendations, the four classes of embryos were defined as A, B, C and D, where class A represents the highest quality of embryos [13]. Per the protocol of our clinic, all patients had fresh embryo transfers of 1 to 3 quality embryos (A/B). In all cases, embryo transfer was performed on day three under the ultrasound control.

The ultrasound check-up of patients was performed two and six weeks after embryo transfer, along with human chorionic gonadotropin (HCG) testing. An ultrasound finding of an intrauterine gestational sac and the heartbeat of the embryo at the second gestational month of US scan was confirmation of clinical pregnancy. Registered clinical pregnancy and clinical pregnancy rate per started cycle (PR) were our primary outcomes, while overall and per patient fertilization rate (FR—% fertilized oocytes transformed into two pronuclei), as well as implantation rate (IR—number of gestational sacs/number of transferred embryos) were the secondary study outcomes.

### Statistical Analysis

Descriptive statistics were used to summarize the demographic, biochemical, and clinical characteristics. The fertilization, implantation, and clinical pregnancy rates were calculated as treatment success measures. Differences in the investigated parameters between groups were tested by ANOVA or Kruskal–Wallis χ2 test. Finally, we applied binary logistic regression (uni and multivariable) to test the impact of P4 and other assessed parameters on achieving pregnancy in whole sample and according to treatment groups (GI/GII). Regression analysis (linear and binary logistic/univariable and multivariable) was used to test the impact of P4, as well as other assessed parameters for fertilization, implantation, and pregnancy rate. Using the receiver operating characteristics (ROC) analysis, cut-off levels of serum P4 for better IVF success were determined. Values *p* < 0.05 were accepted as being significant. Analyses were performed using SPSS for Windows version 22 (SPSS, Inc., Chicago, IL, USA). 

## 3. Results

A total of 947 patients who underwent the IVF procedure during the examined period were analyzed. Upon applying the study inclusion criteria, a total of 73 patients with endometriosis were included in the study. These patients had 77 cycles of IVF whose outcomes were evaluated, while 11 cycles were cancelled.

Study Group I comprised 63.6% and control Group II comprised 36.4% of the examined women. The women, on average, were 34.06 ± 3.5 years old. Their mean BMI was 22.65 ± 2.5 kg/m^2^. The majority of women had stage III endometriosis, with an average EFI score of 5.97 +/− 1.8 and ASRM score of 16 to 40. 

The short antagonist protocol was commonly used (40.3%) for ovarian stimulation and the ovarian response was generally adequate (67.5%). However, only 9.1% of all embryos were good quality. Still, pregnancy was achieved in 40.3% of all participating women. 

Detailed descriptive data of the examined patients and IVF cycles according to the examined groups are presented in Table 1 and Table 2. 

Women from the study GI had a higher ASRM score (*p* = 0.001) with more advanced endometriosis stage (*p* = 0.001), but also generally more embryos (*p* = 0.031) than the women from the control GII. There were no other significant differences regarding any of the other investigated parameters (age, BMI, basal hormonal status, parameters of IVF, IR, and FR) between the women who were (GI) and were not (GII) treated for endometriosis prior to IVF.

Pregnancy was achieved more often in GI women who were treated for endometriosis before IVF (*p* = 0.040). Women who achieved pregnancy were significantly younger (*p* = 0.027); had a higher EFI score (*p* = 0.031), higher basal serum levels of P4 (*p* = 0.001) and E2 (*p* = 0.002), lower FSH (*p* = 0.015), and higher AMH (*p* = 0.001); were more often stimulated with the long protocol (*p* = 0.027); and had adequate ovarian response (*p* = 0.003) and embryo class (*p* = 0.024) than the women who did not achieve pregnancy. There were no other significant differences in the characteristics of the examined women regarding pregnancy achievement.

Next, we examined the differences in the patient basal hormonal status regarding pregnancy achievement separately for Groups I (treated) and II (not treated). It was seen that in study GI, pregnancy was achieved more often if the basal serum levels of P4 (*p* = 0.026), E2 (*p* = 0.002), and AMH (*p* = 0.001) were higher. In the control GII, pregnancy was achieved more often if the basal serum levels of P4 (*p* = 0.001) and AMH (*p* = 0.001) were higher. The basal serum levels of other the examined hormones showed no significant differences in either of the groups.

In the univariant logistic regression, we found that in endometriosis patients, higher basal serum P4 levels were associated with achieving pregnancy through IVF (R^2^ = 0.522; *p* = 0.001; variance = 70.1%). Moreover, the serum P4 levels were associated with achieving pregnancy both in patients treated for endometriosis (R^2^=0.731; *p*=0.021; variance=65.3%) before IVF and in those who were not treated (R^2^ = 0.506; *p* = 0.001; variance = 89.3%). Higher serum P4 levels were also associated with good implantation and fertility rates in the whole sample, as well as in the IR of the treated patients and FR of the patients non-treated for endometriosis before IVF (Table 3).

Different models were obtained for the prediction of pregnancy achievement, as well as prediction of the implantation rate adjusted for patients and IVF characteristics in the whole sample as well as the study GI and control GII. For all models examined parameters were divided into three coherent groups (hormonal status, patients and IVF data) and in that manner tested as independent predictive variables. For better clarity we opted only to present significant models obtained for the whole sample (Table 4 and Table 5). No significant models were achieved for FR (whole sample and groups). 

Based on the obtained models, higher basal serum levels of P4 were found to be predictors of pregnancy achievement, regardless of endometriosis treatment before IVF. Moreover, lower basal serum levels of FSH, higher levels of LH and AMH, and a lower EFI score could also positively impact pregnancy achievement. This implies that the patient hormonal status and extent of endometriosis are more important than the characteristics of IVF procedures for pregnancy achievement. 

Regarding the endometriosis patients treated prior to IVF, the predictors of pregnancy achievement were basal serum levels of P4 (*p* = 0.042); FSH (*p* = 0.017), LH (*p* = 0.038), and AMH levels (*p* = 0.006); and the use of the long stimulation protocol (*p* = 0.034). In the control group of women who were not treated for endometriosis before IVF, only higher basal serum levels of P4 (*p* = 0.013) indicated a better pregnancy achievement (Table 4).

When the influence of the patient and IVF characteristics on IR were assessed, it was seen that higher basal serum levels of P4 and AMH were associated with better IR in women with endometriosis, regardless of whether or not they had the treatment before IVF (Table 5).

In the group of women treated for endometriosis before IVF, a better IR was associated with higher basal serum levels of P4 (*p* = 0.016) and lower FSH (*p* = 0.023), lower BMI (*p* = 0.010), and the endometriosis stage (*p* = 0.038). Conversely, predictors of pregnancy achievement for endometriosis patients who were not treated before IVF were basal serum levels of FSH (*p* = 0.048) and AMH (*p* = 0.007), but not of P4 (0 = 0.524). 

Finally, the ROC analysis showed that in our whole sample of women with endometriosis, regardless of therapy, pregnancy after IVF occurred more often if the P4 serum levels were above 0.695 ng/mL (sensitivity = 71.0%; specificity = 69.6%; area under the curve = 71.9%; *p* = 0.001). In the group of endometriosis patients treated before IVF, pregnancy occurred more often if the basal P4 serum levels were above 0.695 ng/mL (sensitivity = 70.8%; specificity = 64.0%; area under the curve = 67.6%; *p* = 0.035). For women with endometriosis who were not treated before IVF, the cut-off level of P4 for the prediction of pregnancy was 0.70 ng/mL (sensitivity = 71.4%; specificity = 76.2%; area under the curve = 78.9%; *p* = 0.024). 

## 4. Discussion

Endometriosis has been associated with poorer IVF outcomes, including decreased oocyte retrieval, as well as lower implantation and pregnancy rates [13]. In this study, we emphasized the significance of serum P4 at assessing the outcome of IVF procedures. One of the study novelties were the constructed models for IVF success prediction in endometriosis patients. In addition, this study, for the first time, set the cut-off value of the basal P4 serum level for pregnancy achievement in our population of endometriosis patients. Moreover, this might be significant for selecting the proper personalized stimulation protocol in IVF cycles.

Basal serum P4 appeared to be a predictor of successful outcomes, both in the whole sample of examined endometriosis patients and in the study groups (surgically treated vs. not treated). FR and IR were both positively correlated with serum P4 levels in the cohort, but IR was associated with positive outcomes only in the group of treated patients, and FR was associated in the untreated group. One of the specific pathophysiological mechanisms in endometriosis is progesterone resistance, which is determined by changes in the progesterone receptor (PR) composition. The PR-β isoform expression is decreased and the ER-α isoform is elevated, leading to implantation failure [14]. In patients with minimal and mild endometriosis, peritoneal fluid may contain factors that compromise ovarian steroidogenesis and reduce P4 release [15,16]. It can be expected that in the advanced stages of endometriosis, the P4 levels would be significantly reduced. Decreased progesterone may alter the fine balance between metalloproteinases and tissue inhibitors, requiring supplementation in women with endometriosis undergoing IVF [17,18]. We determined that in our population, the basal serum progesterone threshold that may help in the prediction of IVF success could be around 0.7 ng/mL. Studies regarding the cut-off levels of basal P4 are lacking, and the only available cut-offs for P4 serum levels are those on the day of HCG in order for adequate timing of embryo transfers [19].

The multivariate logistic regression performed in this study showed that the main predictors for favorable IVF outcomes were basal hormonal status, including P4 and AMH levels. The same was for our group of patients treated before IVF, suggesting that the treatment of endometriosis may lead to the improvement in progesterone resistance. On the contrary, in our group of untreated patients, P4 did not emerge as a predictor. Other hormones were more significant for the prediction of pregnancy achievement. This means that P4 appeared to be a predictor of a positive IVF outcome in the endometriosis-treated patients, but not in the untreated ones. As progesterone resistance is presented with the down regulation of the progesterone receptor in endometriosis, one explanation of our findings could be that the treatment of endometriosis could improve the expression of the progesterone receptor, leading to improved implantation, with possible changes in the basal P4 serum levels. This hypothesis should be further thoroughly tested with larger samples.

In some studies, AMH has been proven to be an important prognostic parameter in both treated and untreated patients because of endometriosis [20,21]. Although some studies have not shown a correlation between AMH and endometriosis, other investigations have indicated that significantly lower AMH levels could be found in women with more severe forms of the disease [20,21]. An ovary with endometrioma has a faster decreased in ovarian reserve compared with a healthy ovary (role of endometrioma per se). The presence of endometrioma itself does not reduce the number of obtained oocytes, but the ovarian response may be reduced due to its size or bilateralism, or due to previous surgical treatment, disease recurrence, or the age of the patient [21]. Our results are in line with the findings in the literature, where AMH is emphasized as a parameter that is important for reproductive outcome prediction. In the group of previously treated patients, this might have indirectly shown the effectiveness of the applied treatment. The levels of AMH in untreated patients are usually correlated with the extent of the endometriosis or its impact on the functionality of the active ovarian tissue [22,23]. 

Surgery for endometriosis, without a proper indication, is not recommended [24,25]. Some other authors have found that in women with ovarian endometriosis, in situ ICSI can be safely and successfully performed without prior surgical treatment, as the ICSI outcome has been found to be similar in women who were not-treated compared with those who had laparoscopic cyst stripping before ICSI. Therefore, these authors suggest a more conservative strategy against surgical treatment for endometriosis-related infertility patients in order to avoid any negative effect of surgery on ovarian reserve [26]. Surgery could be considered only if IVF procedures might be positively affected, and if the continuity of the treatment is indispensable. Additional therapy in the form of prolonged pituitary downregulation, optimally before IVF, may increase PR [27]. Prolonged down regulation of the hypothalamic–pituitary–gonadal axis by GnRH agonists (once a month for three months) can lead to an adequate size of antral follicles and normal AMH levels four to eight weeks after therapy [28]. Interestingly, in our study, protocol selection emerged as one of the predictive factors for favorable IR in the group of patients treated for endometriosis before IVF, but not for patients who were not treated for endometriosis. The importance of GnRH agonists in the treatment of endometriosis is indispensable, which indicates that the utilization of a long protocol in the category of patients treated for endometriosis and with favorable levels of P4, AMH, and FSH may lead to favorable outcomes [23]. 

Finally, the literature indicates that IVF pregnancies are most often achieved in patients younger than 35, with an infertility duration of up to 3 years. An EFI score that incorporates history data and disease severity has also been proven as a useful tool for assessing infertility treatment success [11,12]. In our sample of endometriosis patients, besides P4 serum levels, a higher EFI score was also found to be a significant predictor of a successful IVF outcome. 

The strength of this study was the fact that it points out the importance of continuity in endometriosis treatment prior to IVF, regardless of its stage. Moreover, the study emphasized the individualization of treatment in endometriosis-related infertility patients in everyday clinical practice. In addition, this prospective study presented findings that are based on a thorough statistical analysis. 

Several study limitations should be mentioned. First, the major limitation of the study is the small sample size, which did not allow for dividing patients and for further analysis in different groups based on the categories of the examined patients (age, BMI, etc.) and the IVF characteristics. The final sample was considerably smaller than the overall number of patients who were submitted to IVF in our clinic during the study period. However, to overcome any potential confounding effects on the IVF outcome, we set strict inclusion criteria to investigate only the outcome of IVF in patients with primary infertility due to endometriosis, and without any other associated infertility factors. Second, as a criterion for surgical treatment (cyst size), we used ESHRE recommendations, but with the possibility for selection bias. Third, a major bias could come from the fact that we analyzed different stimulation protocols in IVF cycles. Nevertheless, all prediction models were adjusted for protocol type, and this was a confounding factor only in patients treated for endometriosis and not in the control group or in the whole sample. Moreover, even after splitting the patients according to the stimulation protocol, basal P4 was confirmed to be a good predictor of IVF success. Fourth, a number of other variables were found to influence IVF success in the regression analysis. However, the investigated parameters were tested, besides P4, in the regression analysis as confounding factors, which provided the preliminary results of their potential impact on IVF success in endometriosis patients. Next, according to the Bologna Criteria for assessing ovarian response to controlled stimulation, all of the examined women had a good ovarian response [29]. However, the Bologna Criteria were not considered for further analysis, as in our study, the assessment was actually based on the study inclusion criteria and not on the patient condition. In our study, all women were 40 years old or younger, had no other risk factors for a poor ovarian response, and had a good ovarian reserve. Finally, the clinical application of the obtained cut-off value of the basal P4 could be debatable, as basal P4 levels are not routinely considered in most institutions. As a consequence, the relevance of the present findings is still limited and needs further assessment. However, precisely because P4 is not routinely examined before IVF in all institutions, the authors of this paper suggest an individualized/personalized approach to patients with endometriosis and infertility that would include the determination of the basal P4 levels for predictive purposes of IVF success. 

Further research incorporating additional parameters that might influence IVF outcome in endometriosis patients, such as the parameters of autoimmune pathologies (B-cell chronic lymphocytic leukemia/lymphoma 6-BCL-6, interleukins, etc.) and their correlations with basal hormonal status, should be performed [30,31]. 

## 5. Conclusions

The serum level of basal progesterone was found to be a reliable predictor of IVF success in patients with endometriosis, regardless of its treatment. Therefore, it is advisable to determine the basal P4 for all endometriosis patients as it could be useful for an individualized approach to infertility treatment and for the prediction of IVF outcomes. The cut-off value of the basal P4 serum levels for IVF success in endometriosis patients was determined in this study for the first time according to available the literature. Based on our study results, the basal serum P4 level should be ≥0.7 ng/mL for successful IVF. However, if endometriosis treatment was taken into consideration, basal P4 serum levels were better indicators of IVF success for patients treated for endometriosis before the procedure than for those directly subjected to IVF. This indicates that comprehensive treatment of endometriosis can lead to improved progesterone resistance and more favorable IVF outcomes. Constructed models for IVF success prediction point out the importance of measuring the basal P4 serum levels, through which personalized treatment for infertility as a result of endometriosis can be planned. 

## Figures and Tables

**Table 1:** Descriptive parameters of the investigated endometriosis patients according to the examined groups.

Parameters	Study Group I (Treated)		Control Group II (Not Treated)		Pregnancy		No Pregnancy	
	Mean	SD	Mean	SD	Mean	SD	Mean	SD
Patients age	33.85	3.18	34.42	3.94	33.06	3.32	34.73	3.43
Body mass index (BMI)	22.65	2.44	22.66	2.57	22.82	2.45	22.54	2.51
Endometriosis fertility index	6.04	1.95	5.85	1.62	6.51	1.85	5.60	1.74
Progesterone (P4)	1.04	0.91	1.02	1.33	1.59	1.38	0.66	0.57
Estradiol (E2)	41.75	21.12	49.42	41.38	54.70	31.76	37.68	27.12
Follicle stimulating hormone	7.42	3.78	6.81	3.23	6.03	3.20	7.98	3.64
Luteinizing hormone	3.99	2.36	3.45	2.29	4.23	2.70	3.50	2.03
Anti-Mullerian hormone	1.36	1.11	1.53	1.14	2.23	1.32	0.87	0.44
Transferred embryo number	1.74	0.23	1.67	0.84	2.05	0.73	1.95	0.36
Fertilization rate (FR)	59.51	25.23	48.59	26.32	52.86	23.96	58.22	27.69
Implantation rate (IR)	21.59	23.74	10.87	30.42	36.83	27.19	/	/
Pregnancy rate per cycle	48.98	22.45	25.00	23.81	93.94	27.66	/	/

**Table 2:** Frequency (%) of the assessed parameters according to the examined groups.

Parameters (%)		Study Group I (Treated)	Control Group II (Not Treated)	Pregnancy	No Pregnancy
Therapy groups	I treated	100	/	77.4	54.3
	II not treated	/	100	22.6	45.7
EM stage	I	2.0	32.1	9.7	15.2
	II	6.1	14.3	9.7	8.7
	III	51.0	42.9	48.4	47.8
	IV	40.8	10.7	32.3	28.3
ASRM score	under 16	8.2	46.4	19.4	23.9
	16 to 40	51.0	42.9	48.4	47.8
	41 to 70	28.6	7.1	22.6	19.6
	71 and more	12.2	3.6	9.7	8.7
Protocol type	short AG	28.6	21.4	16.1	32.6
	short antaG	40.8	39.3	35.5	43.5
	long AG	30.6	39.3	48.4	23.9
Used gonadotropins	FSH	38.8	46.4	54.8	32.6
	HMG	8.2	28.6	9.7	19.6
	FSH+HMG	53.1	25.0	35.5	47.8
Ovarian response	poor	12.2	17.9	25.8	56.5
	adequate	63.3	75.0	58.1	41.3
	excessive	24.5	7.1	16.1	2.2
Embryo class	no embryos	51.0	75.0	0	23.9
	adequate	10.2	7.1	61.3	50.0
	inadequate	38.8	17.9	38.7	26.1
Pregnancy	no	51.0	75.0	/	100
	yes	49.0	25.0	100	/

EM—endometriosis; AG—agonist; antaG—antagonist; FSH—follicle stimulating hormone; LH—luteinizing hormone; HMG—human menopausal gonadotropin; ASRM—American Society of Reproductive Medicine.

**Table 3:** Unadjusted models of the relationship between the P4 levels and pregnancy achievement.

Parameters			B Coefficient	Wald Coefficient	Odds Ratio	*p*	Lower 95% CI	Upper 95% CI
Whole sample	Preg yes/no	P4 level	1.104	10.199	3.017	0.001	1.532	5.942
		Constant	−1.494	13.748	0.225	0.001		
Group I study-treated		P4 level	0.806	4.310	2.239	0.038	1.046	4.793
		Constant	−0.854	3.253	0.425	0.071		
Group II control- not treated		P4 level	1.683	4.436	5.382	0.035	1.124	25.772
		Constant	−2.866	10.196	0.057	0.001		
Parameters			Unstandard B	Standard error	Standard B	*p*	Lower 95% CI	Upper 95% CI
Whole sample	FR	Constant	62.320	4.350		0.001	53.630	71.011
		P4 level	−5.968	2.746	0.262	0.033	−11.453	−0.483
	IR	Constant	10.062	4.357		0.024	1.358	18.765
		P4 level	6.524	2.750	0.284	0.021	1.032	12.017
Study Group I (treated)	FR	Constant	61.870	5.900		0.001	49.954	73.786
		P4 level	−2.172	4.081	−0.083	0.597	−10.413	6.069
	IR	Constant	9.030	5.157		0.047	−1.386	19.446
		P4 level	9.327	3.567	0.378	0.012	2.124	16.531
Control Group II (not treated)	FR	Constant	58.923	6.308		0.001	45.806	72.041
		P4 level	−9.010	3.474	−0.492	0.017	−16.235	−1.784
	IR	Constant	8.904	8.206		0.290	−8.161	25.970
		P4 level	4.240	4.520	0.201	0.359	−5.160	13.641

IR—implantation rate; FR—fertility rate; preg—pregnancy; P4—progesterone; CI—confidence interval; standard—standardized.

**Table 4:** Multivariable regression of the relationship between P4 levels and pregnancy achievement.

Parameters		B Coefficient	Wald Coefficient	*p*	Odds Ratio	Lower 95% CI	Upper 95% CI
Whole sample-hormons model	P4 level	1.172	4.601	**0.032**	3.227	1.106	9.413
	E2 level	0.002	0.034	0.853	1.002	0.979	1.026
	FSH level	−0.292	5.052	**0.025**	0.747	0.579	0.963
	LH level	0.527	5.410	**0.020**	1.694	1.086	2.640
	AMH level	1.976	13.866	**0.001**	7.214	2.550	12.412
Whole sample-patient data model	P4 level	1.378	10.877	**0.001**	3.968	1.749	9.001
	Age	−0.076	0.615	0.433	0.926	0.766	1.121
	BMI	0.111	0.930	0.335	1.118	0.891	1.402
	EFI score	0.528	5.722	**0.017**	1.696	1.100	2.614
	ASRM score	0.643	0.663	0.416	1.903	0.404	8.953
	EM stage	−0.096	0.020	0.888	0.908	0.237	3.474
Whole sample-IVF data model	P4 level	1.093	8.061	**0.005**	2.984	1.403	6.348
	Protocol	0.400	1.033	0.309	1.492	0.690	3.225
	Used GT	−0.343	1.119	0.290	0.710	0.376	1.339
	Ovarian resp	0.783	2.436	0.119	2.189	0.818	5.855
	ET number	0.795	0.573	1.923	0.165	2.214	0.720
	Embryo class	0.523	1.207	0.272	1.686	0.664	4.283

P4—progesterone; E2—estradiol; FSH—follicle stimulating hormone; LH—luteinizing hormone; AMG—anti-Mullerian hormone; BMI—body mass index; GT—gonadotropins; resp—response; ET—embryo transfer; EFI—endometriosis fertility index; ASRM—American Society of Reproductive Medicine; EM—endometriosis; standard—standardized; CI—confidence interval.

**Table 5:** Multivariable regression of the relationship between P4 levels and the implantation rate.

Parameters		Unstandard B	Standard Error	Standard B	*p*	Lower 95% CI	Upper 95% CI
Whole sample-hormons model	P4 level	4.918	3.352	0.214	**0.048**	−1.788	11.623
	E2 level	−0.090	0.118	−0.109	0.446	−0.326	0.145
	FSH level	−0.155	1.537	−0.014	0.920	−3.229	2.918
	LH level	−2.821	3.045	−0.132	0.358	−8.912	3.270
	AMH level	7.800	2.757	0.350	**0.006**	2.285	13.314

P4—progesterone; E2—estradiol; FSH—follicle stimulation hormone; LH—luteinizing hormone; AMG—anti-Mullerian hormone; BMI—body mass index; GT—gonadotropins; resp—response; EFI—endometriosis fertility index; ASRM—American Society of Reproductive Medicine; EM—endometriosis; standard—standardized; CI—confidence interval.

## Data Availability

The data presented in this study are available upon request from the corresponding author, but are not publicly available as there are personal patients’ findings.
